# Overweight and obese pre-pregnancy BMI is associated with higher hospital costs of childbirth in England

**DOI:** 10.1186/s12884-018-1893-z

**Published:** 2018-06-20

**Authors:** Francesca Solmi, Stephen Morris

**Affiliations:** 1 Department of Applied Health Research, University College London, London, UK; 2 Division of Psychiatry, University College London, 6th Floor, Wing B, Maple House, 149 Tottenham Court Road, London, W1T 7NF UK

**Keywords:** Pregnancy, Hospital costs, Obesity, Pre-pregnancy BMI, Pregnancy complications, Length of stay

## Abstract

**Background:**

Women who have an overweight or obese BMI are more likely to experience pregnancy complications. However, little is known on the cost of childbirth in this group and no studies have been undertaken in England to date. The aim of this paper is therefore to investigate whether women with overweight and obese pre-pregnancy body mass index (BMI) incur higher average hospital costs of childbirth.

**Methods:**

We employed data from 7564 women in the first wave of data collection of the Millennium Cohort Study. Using interval regression, we investigated the association between hospital costs of childbirth and pre-pregnancy BMI, fitting four models, progressively adjusting for additional potential confounders and mediators. Model 1 was a univariate model; model 2 adjusted for maternal age, education, marital status, ethnicity, income, and region; model 3 additionally included number of previous children, number of babies delivered, whether birth was at term, and type of delivery; model 4 also included length of hospital stay.

**Results:**

Childbirth costs incurred by women who were overweight, obese class I and obese class II and III were £22, £82 and £126 higher than those incurred by women whose BMI was in the normal range (*p* ≤ 0.05). Delivery method, pre-term delivery, and length of hospital stay accounted for the observed difference.

**Conclusions:**

Women with elevated pre-pregnancy BMI make greater use of services resulting in higher hospital costs. Interventions promoting healthy BMI in pre-pregnancy among women of child-bearing age have the potential to reduce pregnancy complications and be cost-effective.

**Electronic supplementary material:**

The online version of this article (10.1186/s12884-018-1893-z) contains supplementary material, which is available to authorized users.

## Background

In the past two decades concern has mounted over the increasing prevalence of obesity among women of child-bearing age in most developed countries [1, 2]. In England, the prevalence of obesity in the first trimester of pregnancy has risen from 7.6 to 15.6% between 1989 and 2007 [3].

Growing evidence suggests that high levels of pre-gravid body mass index (BMI) are associated with poor pregnancy and birth outcomes [4, 5]. Elevated BMI is believed to increase maternal risk of developing pre-eclampsia [6, 7], gestational diabetes [7], as well as of experiencing still-birth, and neonatal, perinatal, and infant death [8–10]. Compared to women whose BMI is in the normal range, women who are obese have higher odds of complications at delivery such as macrosomia [9], post-term and induced deliveries, and Caesarean sections [8–10].

In stark contrast with the wealth of epidemiological evidence on the negative outcomes associated with obesity in pregnancy, little attention has been paid to quantifying their cost. To date, only five studies have investigated differences in the cost of childbirth between mothers with normal and overweight/obese BMI [11–15]. In spite of the heterogeneity in regional settings and health systems (Australia [13]; United States [14, 16]; France [12, 15]), all studies found that mothers who were obese incurred higher hospital costs compared to mothers with normal BMI and that mode of delivery (i.e. caesarean section) [12, 14, 15], gestational diabetes mellitus [16], and length of stay (LOS) were responsible for the higher cost [12–15].

To the best of our knowledge, no studies have investigated the association between pre-pregnancy BMI and hospital costs of childbirth in England. Therefore, in this paper we aim to do so using general population data from the Millennium Cohort Study. We hypothesised that women with higher BMI would incur higher costs of childbirth.

## Methods

### Sample

The Millennium Cohort Study (MCS) is a prospective cohort of 18,818 children (18,296 singletons, 246 twins, and 10 triplets) who were born between 1st September 2000 and 11th January 2002, resided in the United Kingdom (UK) at 9 months, and were eligible for receiving Child Benefits (universal benefit payable to families who are permanent UK residents) [17]. The cohort includes children living in non-household situations and children who were not born in the UK, but lived in the UK at recruitment. A stratified clustered framework was employed to ensure adequate representation of disadvantaged and ethnic minority groups [17].

Supplementary clinical and demographic data on the participating families and babies were obtained by linking MCS data with birth registration records available from the Office for National Statistics (ONS) as well as with Maternity Hospital Episodes Statistics (HES) records. Linking was successful for 89% (99% of those who gave consent) of MCS babies in the case of birth registration data and in 74% (83% of those who gave consent) of cases for HES data [18].

In this study, we employed linked data on 10,184 English births (singletons only), as data on total hospital costs was only available for England [18].

### Exposure and outcome measures

At the first sweep of data collection (MCS1), when the child was approximately 9 months old, mothers were asked: “Thinking back to just before you became pregnant with [baby’s name], what was your weight then (without clothes)?” From this measure of weight and self-reported height, we derived pre-pregnancy body mass index (=Kilograms/metres^2^, BMI), which we subsequently categorized as: underweight (< 18.5), normal weight (18.5–24.5), overweight (25.0–29.9), obese class I (30.00–34.99), and obese class II and III (≥35.00). The obese classes II and III were grouped together due to low numbers in each group.

Data on average cost of child birth was derived from HES records by calculating adding hotel cost per day for the mother’s length of stay (LOS) to the cost of delivery (i.e., cost of delivery + hotel cost per day*LOS) Cost of delivery is derived by applying national average unit cost to women’s mode of delivery, procedures undertaken, and anaesthesia during delivery and post-delivery [19]. In the MCS costs are reported in £250 bands (e.g. 0-£250; £250–£500) using 2000–2002 prices. Cost figures reported in the MCS dataset are similar to the reference cost calculation (average cost per delivery: £749, range £161 -£4656) for the same year (2000–2001).

### Other variables

We employed data on several maternal socio-demographic characteristics: age; highest academic qualification (no education, GCSE/A-levels, higher education, other); ethnicity (White, Mixed, Indian, Pakistani or Bangladeshi, Black Caribbean (including Black British) or Black African, other ethnic group); OECD equivalised income quintile (1 = most deprived; 5 = least deprived); marital status (single parent or living as a couple); and Government Office Region of residence (North East, North West, Yorkshire and the Humber, East Midlands, West Midlands, East of England, London, South East, South West, Wales, and Northern Ireland).

In addition, we included data on: number previous pregnancies; method of delivery (natural birth; forceps or vacuum extraction or breech; elective or emergency caesarean section); whether the birth was pre-term (i.e. < 37 weeks of gestation, yes/no and episode (i.e. the time spent under the care of one consultant) duration in days.

Data on household income, education, ethnicity, type of pregnancy (single or multiple), number of previous pregnancies, and marital status were self-reported. Data on region, maternal age, and episode duration and cost were obtained from HES data. Data on method of delivery were obtained from HES data and supplemented by self-report data when the former was missing (15% cases). Where more than one method of delivery was reported in the self-reported data (1.76% cases), we assigned the most intensive method to that birth (caesarean section then induced pregnancy, then spontaneous birth).

### Data analyses

The MCS sampled participants by electoral ward employing a stratified clustered sampling framework aimed at achieving a sample which was representative of all four UK countries, and of disadvantaged and ethnic minority groups. In order to produce unbiased estimates, we employed cross-sectional survey weights, accounting for sampling strata and non-response, with finite population correction [20].

We used cross-tabulations with Chi-square tests and analysis of variance (ANOVA) to describe the sample with respect to the covariates included in the analyses and to run descriptive associations between the covariates and maternal pre-pregnancy BMI category.

In order to account for the banded nature of the cost variable when exploring the association between childbirth costs and maternal pre-pregnancy we used interval regression as our data did not appear excessively skewed upon visual inspection (Supplemental Figure1, see Additional file 1: Figure S1). As a sensitivity analyses, we also ran our analyses using ordinal probit model (more appropriate for skewed data) and obtained comparable results in terms of direction, size, and significance of the association. The qualitatively similar findings between the ordered probit and interval regression models is reassuring as the former is less prone to problems arising from skewed data. Given this reassurance, in the paper we present the interval regression results as these better account for the cardinal nature of our dependent variable.

We ran 4 models, as follows: Model I was unadjusted – we included no covariates; in model II we adjusted for maternal age, education, marital status, ethnicity, equivalised income, and region; in model III we adjusted for all the covariates in model II plus number of previous children, number of babies delivered, pre-term birth, and type of delivery; and in model IV adjusted for all the covariates in model III plus we included LOS as a potential moderating factor. Model III and IV were included in order to investigate factors associated with increased costs and whether they explained any observed association between BMI and the outcome.

We only included mothers with complete data on all variables of interest, but compared women with missing covariate data to those with complete data. All analyses were run using Stata13 [21]. We report B coefficients and 95% confidence intervals for each covariates; the B coefficients are marginal effects, reflecting the impact of each covariate on childbirth costs in UK pound.

## Results

### Sample

**Fig. 1:**
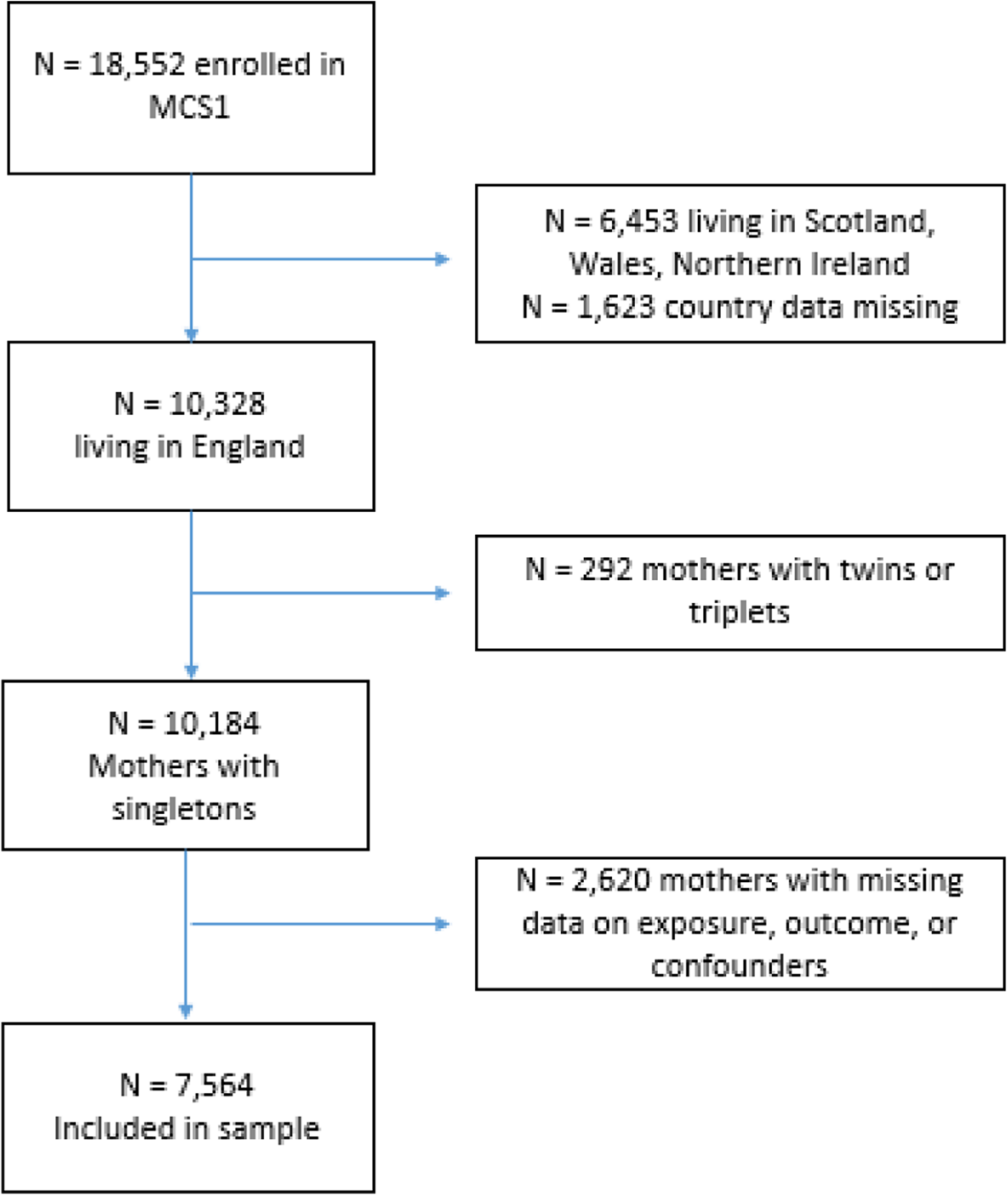
Flowchart of study participation

A total of 10,328 mothers were sampled in the first wave of MCS data collection in England. Of these, 2669 had missing data on hospital costs and covariates and were therefore removed from the analyses. Our final sample consisted of 7564 mothers (73.2% of total) after we excluded women who had given birth to twins or triplets (*N* = 95, 1.24%) (Fig. 1). The majority of the women included in our sample were living as a couple (60.9%), were educated up to GCSE/A-levels (57.5%), were of white ethnicity (90.1%), lived in the Midlands and East of England (30.9%), were primiparae (93.4%), and gave birth at term (93.4%). Mean maternal age was 28.8 (standard error (SE) = 0.2). Mean LOS was 2.8 days (SE = 0.1) (Table 1).

**Table 1:** Sample characteristics and distribution of variables included in regression models across levels of pre-pregnancy BMI (*N* = 7564)

Variables	Total N (%)	Pre-Pregnancy Body Mass Index					*P*-value (Χ^2^)
		Underweight N (%)	Normal weight N (%)	Overweight N (%)	Obese Class I N (%)	Obese Class II & III N (%)	
Total	7564 (100.0%)	473(5.5%)	4897(66.0%)	1502(19.5%)	488(6.4%)	204(2.6%)	
Education							
None of these	1465 (15.4%)	163 (31.9%)	873 (13.9%)	305 (15.8%)	85 (13.9%)	39 (17.9%)	< 0.0001
GCSE/A level	4260 (57.5%)	244 (54.1%)	2714 (56.2%)	866 (59.5%)	306 (64.9%)	130 (64.1%)	
Higher education	1839 (27.1%)	66 (14.0%)	1310 (29.9%)	331 (24.7%)	97 (21.2%)	35 (18.0%)	
Marital status							
Couple	4545 (60.9%)	205 (38.7%)	2934 (61.2%)	946 (63.2%)	330 (68.1%)	130 (65.9%)	< 0.0001
Single	3019 (39.1%)	268 (61.3%)	1963 (38.8%)	556 (36.8%)	158 (31.9%)	74 (34.1%)	
OECD equivalised income quintile							
1st (lowest)	1570 (16.5%)	185 (34.92%)	979 (15.6%)	294 (19.6%)	73 (15.0%)	41 (16.4%)	< 0.0001
2nd	1605 (18.4%)	138 (27.28%)	918 (16.4%)	347 (23.1%)	141 (28.9%)	62 (28.1%)	
3rd	1473 (20.4%)	70 (18.25%)	944 (19.8%)	309 (20.6%)	107 (22.0%)	43 (22.1%)	
4th	1440 (21.2%)	37 (7.69%)	964 (19.7%)	300 (20.0%)	100 (20.5%)	39 (22.4%)	
5th (highest)	1476 (23.5%)	43 (11.33%)	1092 (26.5%)	252 (16.8%)	67 (13.7%)	22 (11.0%)	
Ethnicity							
White	6259 (90.1%)	340 (83.2%)	4137 (91.2%)	1208 (88.7%)	397 (88.4%)	177 (92.7%)	< 0.0001
Mixed/Other	249 (2.5%)	28 (3.7%)	157 (2.4%)	45 (2.1%)	15 (3.0%)	4 (1.4%)	
Indian/ Pakistani/Bangladeshi	794 (5.2%)	88 (9.7%)	471 (4.8%)	176 (6.2%)	45 (4.4%)	14 (2.9%)	
Black Caribbean or Black African	262 (2.2%)	17 (3.4%)	132 (1.6%)	73 (3.0%)	31 (4.2%)	9 (3.0%)	
Government Office Region*							
North	2345 (29.7%)	158 (32.5%)	1523 (29.8%)	460 (29.4%)	139 (27.9%)	65 (28.9%)	0.40
Midlands & East	2391 (30.9%)	138 (27.2%)	1505 (30.3%)	504 (32.0%)	171 (33.9%)	73 (30.9%)	
London	1213 (13.8%)	90 (16.2%)	793 (13.9%)	231 (13.7%)	77 (13.4%)	22 (13.8%)	
South	1615 (25.6%)	87 (24.1%)	1076 (26.0%)	307 (24.9%)	101 (24.8%)	44 (26.5%)	
Premature							
No	7055 (93.4%)	428 (90.4%)	4575 (93.7%)	1419 (94.2%)	457 (93.4%)	176 (85.7%)	0.0002
Yes	509 (6.6%)	45 (9.6%)	322 (6.3%)	83 (5.8%)	31 (6.6%)	28 (14.3%)	
Parity							
Primiparae	7291 (96.5%)	444 (93.6%)	4747 (97.0%)	1445 (96.0%)	466 (95.5%)	189 (93.2%)	0.001
Multiparae	273 (3.5%)	29 (6.4%)	150 (3.0%)	57 (4.0%)	22 (4.5%)	15 (6.8%)	
Notional cost of treatment (UK£)							
1–250	11 (0.2%)	1 (0.2%)	5 (0.2%)	3 (0.2%)	2 (0.6%)	0 (0.00%)	< 0.0001
250–500	1126 (14.9%)	86 (17.7%)	747 (15.7%)	218 (14.1%)	58 (10.9%)	17 (9.2%)	
500–750	3615 (47.7%)	238 (51.8%)	2.394 (48.5%)	695 (47.3%)	198 (39.9%)	90 (42.5%)	
750–1000	1701 (22.7%)	93 (18.7%)	1108 (22.8%)	331 (22.4%)	126 (27.0%)	43 (20.2%)	
1000–1250	656 (8.8%)	28 (5.9%)	385 (8.0%)	151 (9.8%)	59 (12.4%)	33 (18.6%)	
1250–1500	221 (2.8%)	12 (2.2%)	126 (2.5%)	54 (3.3%)	26 (5.5%)	3 (1.1%)	
1500–1750	85 (1.1%)	5 (1.1%)	46 (0.9%)	21 (1.3%)	6 (1.2%)	7 (3.1%)	
1750–2000	43 (0.6%)	2 (0.5%)	21 (0.4%)	12 (0.8%)	3 (0.5%)	5 (3.0%)	
2000 or over	106 (1.2%)	8 (1.8%)	65 (1.2%)	17 (0.9%)	10 (2.0%)	6 (2.4%)	
	Mean (SE)	Mean (SE)	Mean (SE)	Mean (SE)	Mean (SE)	Mean (SE)	Mean (SD)
Maternal age (years)	28.8 (0.2)	25.0 (0.3)	28.8 (0.2)	29.4 (0.2)	29.3 (0.3)	29.8 (0.4)	< 0.0001
Length of stay (days)	2.8 (0.1)	2.6 (0.2)	2.7 (0.1)	2.9 (0.1)	3.4 (0.2)	3.8 (0.3)	< 0.0001

Abbreviations: *N* Number of women, *SE* Standard error

*North includes: North East, North West, Yorkshire & the Humber, Northern Ireland, and Wales; South Includes: South East and South West; Midland and East include: East Midlands, West Midlands, and East of England

### Missing data

We compared women with complete data on exposure and outcome variables against those with any missing covariates. Women with complete data were more likely to be of white ethnicity, older, primiparae, educated at least to GCSEs/A-level, wealthier, have lived in the south and north east regions of England, and have given birth at term. We did not find any differences with respect to the other covariates.

### Maternal pre-pregnancy BMI

The majority of women in our sample had a normal BMI before pregnancy (*N* = 4897, 66.0%); 473 (5.5%) were underweight, and 1502 (19.5%), 488 (6.4%), and 204 (2.6%) were overweight, obese (class I), and obese (class II and III) respectively (Table 1). Compared to normal weight and underweight mothers, a greater proportion of mothers who were overweight and obese had low levels of education, were single, were of Pakistani/Bangladeshi, or Black Caribbean or Black African ethnicity and were older. Women who were obese had a higher proportion of pre-term deliveries compared to normal weight women, but not compared to underweight women (Table 1).

### Regression models

In the unadjusted model (model I) mothers who were overweight and obese (both categories) had higher childbirth costs compared to normal weight mothers (overweight = B coefficient (coeff): 21.88, 95% Confidence Interval (CI):4.46; 39.29; obese (class I) = B coeff: 81.59, 95% CI: 48.37; 114.80; obese (class II and III) = B coeff: 126.41 95% CI:63.51; 189.31). In model II the direction, strength, and magnitude of the univariate association between maternal BMI and childbirth costs remained substantially unchanged (overweight = B coeff: 23.62, 95% CI: 5.68; 41.57; obese (class I): B coeff: 86.01 95% CI: 53.64; 118.37; obese (class II and III) = B coeff: 134.13, 95% CI: 72.78; 195.47). In model III, although the strength and direction of the associations were retained, the magnitude was reduced for women who were obese class I (B coeff: 43.29, 95% CI: 14.39; 72.19) and obese class II and III (B coeff: 55.22, 95% CI: -2.02; 112.46) whilst the association was no longer significant for overweight mothers (B coeff; 9.79, 95% CI: -5.11; 25.00) (Table 2).

**Table 2:** Univariate and multivariate interval regression models for the association between maternal pre-pregnancy Body Mass Index and hospital costs of childbirth (*N* = 7564)

	Total cost of child birth			
	Model I Coefficient (95%CI)	Model II Coefficient (95%CI)	Model III Coefficient (95%CI)	Model IV Coefficient (95%CI)
Pre-pregnancy BMI				
Underweight	−14.84 (−48.04; 18.34)	0.78 (−32.64; 34.19)	−7.85 (− 34.32; 18.61)	− 4.09 (− 17.15; 8.97)
Normal weight	Ref.	Ref.	Ref.	Ref.
Overweight	21.88 (4.46; 39.29)**	23.62 (5.68; 41.57)**	9.79 (−5.11; 25.00)	−2.92 (−10.55; 4.70)
Obese (class I)	81.59 (48.37; 114.80)**	86.01 (53.64; 118.37)**	43.29 (14.39; 72.19)**	−0.37 (−12.25; 11.51)
Obese (class II and III)	126.41 (63.51; 189.31)**	134.13 (72.78; 195.47)**	55.22 (−2.02; 112.46)*	−1.64 (− 25.03; 21.74)
Education				
None of these	–	Ref.	Ref.	Ref.
GCSE/A level	–	19.76 (−0.34; 39.87)*	12.94 (−4.37; 30.26)	−3.36 (−12.29; 5.57)
Higher education	–	41.53 (15.18; 67.89)**	25.55 (3.62; 47.49)**	−0.82 (−11.02; 9.38)
Marital Status				
Single	–	24.89 (7.53; 42.23)**	20.81 (5.28; 36.34)**	1.49 (−6.06; 1.11)
Couple	–	Ref.	Ref.	Ref.
Maternal Age	–	1.32 (−0.28; 2.92)	−0.72 (−2.09; 0.65)	0.52 (− 0.08; 1.11)
OECD equivalised income quintile				
1st (lowest)	–	Ref.	Ref.	Ref.
2nd	–	0.81 (−21.95; 23.58)	−2.76 (− 23.18; 17.66)	−1.71 (−10.85; 7.42)
3rd	–	9.48 (−19.06; 38.030	7.90 (−17.77; 33.56)	2.70 (− 5.88; 11.28)
4th	–	34.55 (9.24; 59.86)**	19.55 (−1.30; 40.40)*	−6.17 (− 17.37; 5.02)
5th (highest)	–	56.73 (24.26; 89.21)**	33.96 (4.45; 63.46)**	2.19 (−9.49; 13.87)
Ethnicity^b^				
White	–	Ref.	Ref.	Ref.
Mixed/Other	–	68.75 (13.33; 124.17)**	45.54 (−3.59; 94.67)	3.17 (−13.35; 19.69)
Asian	–	64.51 (31.11; 97.90)**	57.11 (30.49; 83.73)**	−0.25 (−18.38; 17.87)
Black Caribbean or Black African	–	93.14 (41.99; 144.30)**	69.75 (30.85; 108.65)**	16.68 (1.90; 31.46)**
Government Office Region^a^				
North	–	Ref.	Ref.	Ref.
Midlands & East	–	9.33 (−40.88; 59.54)	10.62 (−33.71; 54.96)	8.36 (−9.24; 25.96)
London		−68.82 (−108.87; −28.77)**	−52.61 (−88.24; − 16.97)**	−19.16 (− 49.10; 10.79)
South	–	− 14.49 (− 45.19; 16.22)	− 8.76 (− 37.40; 19.88)	−0.92 (− 21.41; 19.67)
Premature				
No	–	–	Ref.	Ref.
Yes			308.40 (268.12; 348.67)**	−3.53 (−22.06; 15.01)
Type of delivery				
Spontaneous	–	–	Ref.	Ref.
Forceps			129.42 (102.86; 155.98)**	5.40 (−5.33; 16.14)
Caesarean			330.00 (309.58; 350.42)**	10.66 (−1.97; 23.30)*
Other			156.63 (64.70; 248.57)**	34.12 (21.93; 46.31)**
Parity				
Primiparae	–	–	Ref.	Ref.
Multiparae			0.01 (−42; 03; 42.04)	9.88 (−1.71; 21.48)
Length of Stay (days)	–	–	–	116 (112.02; 120.22)**
Constant	732.79 (716.10; 749.48)	641.21 (588.35; 694.07)	619.98 (574.80; 665.17)	407.12 (383.82; 430.39)

The coefficients are also marginal effects

Model I: unadjusted

Model II: adjusted for maternal age, education, marital status, ethnicity, equivalised income, and government region

Model III: Model II + number of previous children, number of babies delivered, pre-term birth, and type of delivery

Model IV: Model III + Length of stay

Abbreviations: *CI* Confidence interval, *BMI* Body mass index, *OECD* Organisation for economic co-operation and development

** *p* ≤ 0.05; * 0.1 ≥ *p* > 0.05

^a^North includes: North East, North West, Yorkshire & the Humber, Northern Ireland, and Wales; South Includes: South East and South West; Midland and East include: East Midlands, West Midlands, and East of England

^b^Asian includes: Indian, Pakistani, and Bangladeshi

In these multivariate models, being single, from the wealthiest income quintiles, of Asian, Black or ethnicity, and from the North were associated with higher costs compared to being in a relationship, being of white ethnicity, and from London. In model II, but not in model III, maternal age was positively associated with childbirth costs. In model III mothers who had a premature delivery, and had an induced delivery, a Caesarean section, or other type of delivery also incurred in higher costs compared to mother who delivered one child, had a pregnancy at term, and had a spontaneous delivery (Table 2).

With the addition of LOS in model IV the coefficients for overweight, obese class I, and obese class II and III pre-pregnancy BMI approached zero and were no longer statistically significant (overweight = B coeff: -2.92, 95% CI: -10.55; 4.70; obese (class I) = B coeff: -0.37, 95% CI: -12.25;11. 51; obese (class II and III) = B coeff: -1.64, 95% CI: -25.03; 21.74). In this model, most of the covariates previously associated with child birth costs were no longer significant, suggesting they were associated with childbirth costs via their impact on LOS. Maternal age, however, again showed a weak positive association with increased costs. (Table 2).

## Discussion

The extent to which maternal obesity is associated with complications of pregnancy and childbirth has been widely investigated, [1, 4–6, 8, 9] but the economic impact on health services of these complications has received relatively little attention [12–15], especially in the UK. In this study, we explored the association between pre-pregnancy BMI and costs of childbirth using rich data from a large cohort study, the Millennium Cohort Study, linked to administrative data on use of maternity services, Hospital Episodes Statistics. Controlling for socio-demographic and socio-economic factors, we found that women with overweight, obese class I, and obese class II and III pre-pregnancy BMI incurred higher childbirth costs, in the region of £22, £82 and £126, respectively.

To the best of our knowledge, this is the first study attempting to quantify the association between maternal obesity and cost of childbirth in England. Previously, a qualitative study on a sample of 33 maternity and obstetric healthcare professionals working in 16 maternity units in National Health Service (NHS) Trusts in the North East of England, had investigated perceptions on the impact of maternal obesity on the provision of health care [22]. The study suggested that obesity in pregnancy represented a condition with high impact on service delivery and costs by means of higher levels of care required, possible complications for both the mother and the child, and higher levels of maternal morbidity [22]. Although in this study we were unable to account for total health care use throughout pregnancy, previous studies provide evidence in support of increased use of inpatient and outpatient services during pregnancy among obese mothers [11] especially if they have co-occurring chronic conditions [16]. Future studies should aim at employing routinely collected data to quantify the total cost of primary and secondary health care use among mothers with elevated pre-pregnancy BMI.

Our findings confirm that longer LOS among mothers who were overweight and obese is likely to be on the causal pathway between maternal BMI and childbirth costs and affects the latter. When we included pre-term births, delivery method, and number of children delivered in the analyses, our estimates were no longer significant for overweight mothers and decreased to £39 to £59 for obese mothers, whilst accounting for LOS removed any differences between BMI groups. These results are broadly in line with findings of previous studies showing that maternal obesity is associated with higher rates of Caesarean sections [14], longer LOS [13, 14], and costs [12–14] compared to normal weight.

The associations appear to remain consistent regardless of the definition of obesity employed and the method by which the latter is ascertained. In their analysis of the US National Impatient Sample (NIS), a routinely collected dataset of inpatient hospital stays, Trasande and colleagues defined mothers as obese based on the presence of a diagnosis of obesity in their medical records. As the authors note, reliance on clinical diagnosis does not allow for a distinction between whether obesity was already present at the start of the pregnancy or due to excessive gestational weight gain (GWG). Use of clinical diagnosis might also lead to under-representing obesity in pregnancy as women with class II or III obesity or with obesity-related comorbidities are more likely to prompt clinicians to enter obesity on the patient’s records [14]. Although this study, by focusing on more severe cases could have resulted in stronger associations, studies using non-clinical populations and self-reported pre-pregnancy BMI have found similar results [12, 13]. In our study we found that women who were overweight and obese had higher childbirth costs, as well as some evidence of a dose-response relationship, with higher costs in Higher BMI categories. This might be indicative of the presence of a dose-response association in clinical outcomes and LOS.

In Model II, only adjusting for socio-demographic and socio-economic characteristics, we found that women with higher income levels and older age had higher childbirth costs (i.e. greater proportions of pregnancy complications and longer LOS), which has previously been documented [23, 24]. Consistent with our results, previous evidence suggest that older mothers of higher socio-economic status (SES) could be at higher risk of pre-term birth and Caesarean section due to higher prevalence of obesity in this group [24]. We also found that single mothers had higher childbirth costs. Previous evidence suggests that although mothers who are in a relationship have higher levels of readiness-for discharge, they tend to have longer LOS compared to single mothers [23]. However, this study did not account for age differences or complications, which could confound this association. Lack of partner support might partially explain longer hospital stays for single mothers. Finally, we found that women of black, south Asian, and other ethnicity had higher childbirth costs and LOS compared to white women, which corresponds to previous findings from England [25]. This study found that women from ethnic minority groups had higher odds of pre-term and low birthweight pregnancy, and that women of black ethnicity were more likely to have a Caesarean section [25]. In fact, in model III, accounting for mode of delivery, type of pregnancy, and gestational week, we found that pre-term birth, methods other than vaginal delivery and number of child per pregnancy were all associated with higher hospital costs, although the association disappeared when LOS was included in model IV.

Our study has several strengths. We employed a large sample of women in England. Data were drawn both from self-reported measurements and linkage with national routinely collected hospital records, and we found high levels of agreement between the two, when both data sources were available for the same measures. Finally, we were able to control for a number of socio-demographic and socio-economic variables as well as for a number of pregnancy characteristics.

A number of limitations should, however, be noted. Pre-pregnancy BMI was self-reported at the first wave of data collection and, thus subject to both recall bias and misreporting. There is evidence that adults tend to overestimate their height and underestimate their weight [26], which, in our study could lead to a proportion of overweight and obese mothers to be misclassified as normal weight and overweight, respectively. This could potentially result in an underestimation of the association under study.

We also did not control for GWG, which has been suggested to both be a risk factor for pregnancy complications [27] and to interact with pre-gravid obesity to worsen (excessive GWG) or moderate (limited GWG) the negative effect of pre-pregnancy obesity on pregnancy outcomes [27, 28]. Future studies should quantify the costs associated with different levels of GWG, as it is an area amenable to prevention and to thus cost-effective interventions.

We only included costs borne by the NHS and we did not account for any indirect costs associated with birth complications and longer LOS. It is possible that longer LOS can be associated with higher social costs, such as transport or productivity costs incurred by family members to attend the woman in hospital. Data for this study were based on costs of childbirth among babies who were born between 1st September 2000 and 11th January 2002. The number of births in England has increased, and the quality and outcomes of maternity services have improved significantly over the intervening period [29]. In addition, during this period, national guidance has become available on weight management before, during and after pregnancy [30]. These factors mean that the costs of maternity services, and the relationship between maternal obesity and these costs, may have changed – our findings may therefore be dated. Further research using more recent data would be beneficial.

Finally, we had a high proportion of missing data (~ 25%) which seemed to be predicted by factors associated with lower SES, therefore suggesting that our sample might not be representative of women in England. However, we cannot infer whether the participants we excluded were systematically different from those we included with similar socio-demographic characteristics with respect to the exposure and the outcome of interest; therefore, we might or might not be under−/overestimating our associations. Moreover, as explained previously, part of the missingness (11%) is explained by factors associated with feasibility of data linkage with HES, a factor which is exogenous to our study.

## Conclusions

We have replicated findings that pre-pregnancy overweight and obesity are associated with greater delivery complications and longer LOS and we have shown that these are responsible for higher cost of childbirth £22, £82 and £126 for overweight, obese class I, and obese class II and III mothers, respectively. This suggests that policies and intervention aimed at curbing obesity among women of child-bearing age could be cost-effective by means of reducing pregnancy and delivery complications, and LOS. Recent studies also found that children born to mothers who were overweight or obese incurred in greater healthcare costs in their first year or life compared to children born to mother who were normal weight [31]. Reducing obesity in women of child bearing age could therefore result in improved health outcomes and lower healthcare costs for their offspring. Future research should therefore attempt to estimate both social and NHS costs associated with obesity throughout pregnancy and the post-partum period as well as the potential for cost-savings associated with interventions aimed at reducing BMI in women of child-bearing age.

## Additional file

Additional file 1: **Figure S1** Distribution of dependent variable. (DOCX 15 kb)

## Abbreviations

ANOVA: ANalysis Of VAriance
BMI: Body mass index
CI: Confidence interval
Coeff: Coefficient
GCSE: General Certificate of Secondary Education
GWG: Gestational weight gain
HES: Hospital Episode Statistics
LOS: Length of stay
MCS: Millennium Cohort Study
NIS: National Impatient Sample
OECD: Organisation for Economic Co-operation and Development
ONS: Office for National Statistics
SE: Standard error
SES: Socio-economic status
