# Supplementary material for: Overweight and obese pre-pregnancy BMI is associated with higher hospital costs of childbirth in England
Source: BMC Pregnancy Childbirth. 2018 Jun 20;18:253. doi: 10.1186/s12884-018-1893-z (PMC6011257; doi:10.1186/s12884-018-1893-z)
Supplement: Supplementary file 1 — Figure S1 Distribution of dependent variable. (DOCX 15 kb) [file 12884_2018_1893_MOESM1_ESM.docx]

Figure 1: Distribution of dependent variable
